# A three-factor nomogram predicts the use of invasive mechanical ventilation within 72 h in preterm infants

**DOI:** 10.3389/fmed.2026.1722043

**Published:** 2026-04-29

**Authors:** Li Guo, Zhiyang Zhang, Ze Zhang, Chunhui Zhao, Huifen Chen

**Affiliations:** 1 Department of Neonatal, The Fourth Hospital of Shijiazhuang, Shijiazhuang, China; 2 Department of Intensive Care Unit, Hebei General Hospital, Shijiazhuang, China

**Keywords:** Apgar score, early-onset sepsis, invasive mechanical ventilation, nomogram, preterm infants

## Abstract

**Background:**

Early identification of preterm infants at risk for invasive mechanical ventilation (IMV) enables timely respiratory support and may reduce ventilation-related harm.

**Objective:**

To develop and internally validate a parsimonious prediction model for IMV within 72 h after birth.

**Methods:**

We conducted a single-center retrospective cohort study (July 2023–June 2024) including 1,059 preterm infants admitted within 72 h of life and randomly split them into training (*n* = 742) and validation (*n* = 317) sets. Exclusions included chorioamnionitis and deaths ≤ 72 h. Forty-five candidate variables were screened; after multiple imputation, least absolute shrinkage and selection operator (20-fold cross-validation, λ_1se_) identified three predictors for multivariable logistic modeling: 1-min Apgar score, pulmonary surfactant administration within 72 h, and early-onset sepsis. The primary endpoint was endotracheal IMV lasting ≥ 12 consecutive hours within 72 h of birth. Discrimination, calibration, and decision-curve analysis (DCA) were assessed. Sensitivity analysis restricted early-onset sepsis to culture-proven cases.

**Results:**

In the validation set, the model achieved an AUC of 0.816; at the optimal probability threshold (0.224), sensitivity, specificity, and accuracy were 0.613, 0.914, and 0.855, respectively. Calibration was good (Brier score 0.096; Hosmer–Lemeshow *P* = 0.28; expected/observed ratio = 1), and DCA showed net benefit across thresholds 0.10–0.70. Culture-proven analysis yielded AUC 0.830 with similar calibration; a pulmonary surfactant × sepsis interaction was significant (β = −2.531, *P* = 0.028).

**Conclusion:**

A three-factor model based on perinatal and early neonatal indicators provides accurate, well-calibrated prediction of IMV within 72 h and is readily implementable for bedside risk stratification; external validation is warranted.

## Introduction

Preterm infants frequently develop respiratory distress syndrome (RDS) due to insufficient endogenous pulmonary surfactant (PS), rapidly progressing to respiratory failure. RDS continues to be a major contributor to mortality and chronic pulmonary complications in extremely preterm infants (1, 2). Although invasive mechanical ventilation (MV) rapidly improves oxygenation during acute episodes, it concurrently poses risks of alveolar overdistension, inflammation, and bronchopulmonary dysplasia (BPD). A recent multicenter prospective study revealed a dose-response relationship between invasive mechanical ventilation duration and BPD risk, where each additional day increases risk by about 13% (3). Hence, immediate identification of high-risk preterm infants at birth and precise determination of optimal respiratory support have emerged as critical management priorities in neonatal intensive care units.

Existing risk assessment tools such as SNAP-II/PE and NeoVent Score generally depend on 6–10 parameters from blood gases, physiological readings, or laboratory tests, necessitating 12–24 h for complete data collection, thus falling short of real-time stratification requirements on the day of delivery (4). Recent machine-learning approaches that integrate continuous monitoring signals have shown promising discrimination for NICU respiratory outcomes (5, 6). Nevertheless, the inherent “black box” nature of these algorithms and frequent data omissions restrict their practical clinical applicability (5, 7). The 2022 European Consensus on RDS emphasizes developing well-validated predictive tools utilizing early perinatal indicators that are readily accessible and easily interpretable. Such tools aim to guide the configuration of non-invasive ventilation and individualized pulmonary surfactant strategies (1). Nevertheless, simplified models concurrently addressing infection status and early PS responsiveness in preterm infants born at < 37 weeks have not been established.

Within routine NICU care, multiple perinatal indicators are captured in the first minutes after birth. Using a retrospective cohort of 1,059 preterm infants, we screened 36 such variables with penalized LASSO regression and multivariable logistic analysis, which retained a parsimonious subset of independent predictors. These predictors were subsequently incorporated into a visual nomogram that was internally validated for estimating the risk of invasive mechanical ventilation within 72 h. Therefore, we aimed to develop and internally validate a parsimonious nomogram for predicting the use of invasive mechanical ventilation within the first 72 h of life in preterm infants.

## Materials and methods

### Study design and participants

The single-center retrospective cohort study was performed from July 2023 to June 2024 at the Neonatal Intensive Care Unit (NICU) of the Fourth Hospital of Shijiazhuang, Hebei Province, China. This unit is a tertiary-level NICU that routinely manages preterm infants requiring non-invasive respiratory support, pulmonary surfactant therapy, invasive ventilation, and continuous cardiorespiratory monitoring. This retrospective chart-review study was approved by the Ethics Committee of the Fourth Hospital of Shijiazhuang (Approval No. 20230134) and written informed consent was obtained from parents or legal guardians. All data were de-identified prior to release to the investigators under a data-use agreement, and authors had no access to any information that could identify individual. Inclusion criteria included: birth gestational age < 37 weeks, admission to NICU within 72 h postpartum, and survival of at least 72 h to ensure sufficient observation of primary outcomes. Exclusion criteria were major congenital malformations, confirmed chorioamnionitis, incomplete critical data, NICU admission after 72 h postpartum, and early palliative care or death.

Chorioamnionitis (histologic or clinically suspected) was excluded because placental histopathology was not routinely available and clinical criteria varied across obstetric teams, increasing risk of exposure misclassification. We also sought to reduce spectrum bias arising from the fetal inflammatory response, which is associated with higher rates of early-onset sepsis and emergent intubation. The resulting model is intended for CA-negative settings; implications for CA-positive cohorts are addressed in the Limitations (8, 9).

Infants who died ≤ 72 h after birth were excluded to avoid immortal-time and competing-risk bias in outcome ascertainment. Our primary aim was to support bedside decisions during the first 72 h among infants who survive the immediate delivery-room period; thus, the model should be interpreted within this target population (5, 10).

### Variable definition and data collection

The primary outcome was the use of invasive mechanical ventilation (IMV)—including conventional mechanical ventilation (CMV), synchronized intermittent mandatory ventilation (SIMV), high-frequency oscillatory ventilation (HFOV), or endotracheal neurally adjusted ventilatory assist (NAVA)—delivered via an endotracheal tube and lasting ≥ 12 consecutive hours within the first 72 h after birth. Non-invasive support (CPAP or NIPPV, including NIV-NAVA) was not counted. For infants briefly intubated solely for surfactant delivery (e.g., INSURE or LISA) and extubated without ≥ 12 h of IMV, the outcome was considered not met. For multiple intubations, timing was anchored to the first IMV episode that met the ≥ 12 h criterion. The 72-h window followed Dargaville et al. (11), and the ≥ 12-h threshold followed Jensen et al. (12).

Our NICU follows a written guideline aligned with the 2023 European Consensus on RDS management. Intubation and initiation of IMV are recommended when any of the following are present despite optimized non-invasive support: (1) FiO_2_ ≥ 0.40 sustained for ≥ 15 min while on nasal CPAP ≥ 8 cmH_2_O; (2) PaCO_2_ > 65 mmHg (≈8.7 kPa) with blood-gas pH < 7.20; or (3) ≥ 4 clinically significant apnea episodes per hour, or any apnea requiring bag-mask ventilation.

Candidate predictors initially comprised 45 perinatal and early neonatal indicators. Variables with ≥ 5% missingness (*n* = 9) were excluded. To ensure analytic integrity, missing data were addressed via multiple imputation using the mice package in R: continuous variables with linear regression and categorical variables with logistic regression. Five imputed datasets were generated, and a sensitivity analysis evaluated model performance across these datasets. Performance metrics (AUC, accuracy, sensitivity, specificity) showed negligible variation, indicating high consistency. Given the minimal differences, Dataset 5 (denoted with an asterisk) was randomly selected for presentation in subsequent analyses (Supplementary Table 1). Candidate predictors and operationalization. After exclusions, 36 analyzable candidates were prespecified. Examples of operationalization include: Apgar at 1, 5, and 10 min assessed in the delivery room (range 0–10); pulmonary surfactant (PS) within 72 h of birth, coded Yes/No and recorded prior to the first IMV episode (brief intubations solely for INSURE/LISA were not counted as IMV events), mirroring the temporal-precedence coding used in a 72-h ventilation-risk model by Yue et al. (10); and early-onset sepsis (EOS ≤ 72 h), defined as culture-proven (positive blood or sterile-site culture) or clinical (≥ 2 compatible signs with laboratory support and ≥ 5 days of IV antibiotics, with no alternative diagnosis); contaminants were excluded. A sensitivity analysis restricted EOS to culture-proven only.

### Data preprocessing and analysis pipeline

Normality of continuous variables was evaluated using the Shapiro–Wilk test, and categorical variables were encoded as binary variables. The dataset was randomly partitioned (set.seed = 3464) into training (*n* = 742) and validation sets (*n* = 317) at a 7:3 ratio. The event-per-variable (EPV) ratio exceeded 10, fulfilling the criteria required for multivariate analysis.

### Statistical analysis

Baseline characteristics: Variables with normal distribution (mean ± SD) were analyzed using independent *t*-tests; non-normally distributed variables (median, IQR) were analyzed using the Mann–Whitney U test; categorical variables were compared using the χ^2^ or Fisher’s exact test. Univariable analysis involved logistic regression to calculate odds ratios (95% CI), *P*-values, and establish a preliminary basis for variable selection (see Supplementary Table 2). Variable selection was performed using LASSO (glmnet, α = 1) with 20-fold cross-validation; we selected λ_1s*e*_ (1-SE rule) to determine non-zero coefficients. A multivariable logistic regression model was constructed based on the LASSO-selected variables, and multicollinearity was assessed using the variance inflation factor (VIF) from the car package, with a threshold of VIF < 2 considered acceptable. Model performance evaluation included discriminative ability assessment via receiver operating characteristic (ROC) curve analysis, reporting the area under the curve (AUC) with 95% CI, and inter-group AUC comparisons using DeLong’s test (pROC package). Calibration was evaluated using 1,000 bootstrap samples to generate calibration curves, accompanied by the Brier score, Hosmer–Lemeshow χ^2^ test, calibration intercept, slope, and expected-to-observed (E/O) ratio. Clinical net benefit was assessed via decision curve analysis (DCA), comparing the developed model with “treat-all” and “treat-none” strategies. Interaction analysis involved creating interaction terms within the multivariable model and visualizing them through interaction plots.

Statistical significance was set at a two-sided α of 0.05. Baseline comparisons and logistic regression analyses (univariable and multivariable) were performed using SPSS version 27.0 (IBM Corp., Armonk, NY, United States), while all other analyses, including LASSO regression, model evaluation, and interaction analysis, were conducted using R version 4.3.2.

### Sensitivity analysis

EOS was redefined as culture-proven only. Using the same train–validation split, we refit the three-predictor logistic model (Apgar at 1 min [per 1-point decrease], PS ≤ 72 h [No PS = 1 vs. PS = 0], EOS) in the training set and evaluated performance in the validation set (ROC/AUC with DeLong CIs, calibration, Brier score, and decision-curve analysis).

## Results

### Baseline characteristics

A total of 1,059 preterm infants were included in the analysis after exclusions (19 with chorioamnionitis and 28 who died ≤ 72 h after birth). Of these, 742 were randomly assigned to the training set and 317 to the validation set. Within the first 72 h after birth, 232/1,059 (21.9%) infants used invasive mechanical ventilation (endotracheal positive-pressure ventilation for ≥ 12 consecutive hours). Baseline characteristics stratified by mechanical-ventilation status are summarized in Table 1 for both sets.

**TABLE 1:** Baseline characteristics of preterm infants in the training and validation cohorts, stratified by mechanical ventilation within 72 h after birth.

Characteristic	Training cohort				Validation cohort			
	All infants	Non-MV	MV	*P*	All infants	Non-MV	MV	*P*
	*N* = 742	*N* = 572	*N* = 170		*N* = 317	*N* = 255	*N* = 62	
Maternal demographics								
Maternal age (years)	31(28, 34)	31(28, 34)	31(28, 34)	0.688	31(28, 34)	31(28, 34)	32(29, 34)	0.090
Maternal occupation				0.178				0.238
No	425(57.3)	320(55.9)	105(61.8)		194(61.2)	152(59.6)	42(67.7)	
Yes	317(42.7)	252(44.1)	65(38.2)		123(38.8)	103(40.4)	20(32.3)	
Maternal smoking				0.999				0.196
No	730(98.4)	562(98.3)	168(98.8)		316(99.7)	255(100)	61(98.4)	
Yes	12(1.6)	10(1.7)	2(1.2)		1(0.3)	0(0)	1(1.6)	
Gravidity (n)	1(1, 2)	1(1, 2)	1(1, 2)	0.883	1(1, 2)	1(1, 2)	1(1, 2)	0.902
Parity (n)	2(1, 2)	2(1, 2)	2(1, 2)	0.542	2(1, 2)	2(1, 2)	2(1, 2)	0.594
Mode of conception				0.602				0.956
No	646(87.1)	500(87.4)	146(85.9)		260(82.0)	209(82.0)	51(82.3)	
Yes	96(12.9)	72(12.6)	24(14.1)		57(18.0)	46(18.0)	11(17.7)	
Maternal complications								
Hypertensive disorders in pregnancy				0.373				0.211
No	535(72.1)	417(72.9)	118(69.4)		225(71.0)	185(72.5)	40(64.5)	
Yes	207(27.9)	155(27.1)	52(30.6)		92(29.0)	70(27.5)	22(35.5)	
Gestational diabetes mellitus				0.507				0.756
No	530(71.4)	412(72.0)	118(69.4)		235(74.1)	190(74.5)	45(72.6)	
Yes	212(28.6)	160(28.0)	52(30.6)		82(25.9)	65(25.5)	17(27.4)	
Intrapartum fever				0.770				0.999
No	726(97.8)	560(97.9)	166(97.6)		309(97.5)	248(97.3)	61(98.4)	
Yes	16(2.2)	12(2.1)	4(2.4)		8(2.5)	7(2.7)	1(1.6)	
Premature rupture of membranes				0.116				0.496
No	532(71.7)	402(70.3)	130(76.5)		224(70.7)	178(69.8)	46(74.2)	
Yes	210(28.3)	170(29.7)	40(23.5)		93(29.3)	77(30.2)	16(25.8)	
Placenta previa				0.365				0.385
No	713(96.1)	547(95.6)	166(97.6)		308(97.2)	249(97.6)	59(95.2)	
Yes	29(3.9)	25(4.4)	4(2.4)		9(2.8)	6(2.4)	3(4.8)	
Placental abruption				0.459				0.999
No	718(96.8)	555(97.0)	163(95.9)		307(96.8)	247(96.9)	60(96.8)	
Yes	24(3.2)	17(3.0)	7(4.1)		10(3.2)	8(3.1)	2(3.2)	
Abnormal placental pathology				0.184				0.121
No	612(82.5)	466(81.5)	146(85.9)		266(83.9)	218(85.5)	48(77.4)	
Yes	130(17.5)	106(18.5)	24(14.1)		51(16.1)	37(14.5)	14(22.6)	
Perinatal characteristics								
Singleton pregnancy				0.132				0.846
No	519(69.9)	408(71.3)	111(65.3)		218(68.8)	176(69.0)	42(67.7)	
Yes	223(30.1)	164(28.7)	59(34.7)		99(31.2)	79(31.0)	20(32.3)	
Neonatal sex				0.159				0.556
Female	419(56.5)	315(55.1)	104(61.2)		153(48.3)	121(47.5)	32(51.6)	
Male	323(43.5)	257(44.9)	66(38.8)		164(51.7)	134(52.5)	30(48.4)	
Gestational age (weeks)	35(34, 36)	35(34, 36)	34(33, 36)	< 0.001	35(34, 36)	35(34, 36)	34(34, 36)	< 0.001
Additional gestational days	3(1, 5)	3(1, 5)	3(1, 5)	0.814	3(1, 5)	3(1, 5)	3(2, 5)	0.666
Birth weight (g)	2370(2028, 2673)	2395(1805, 2395)	2140(1337, 2140)	< 0.001	2380(2035, 2655)	2410(2000, 2580)	2115(1795, 2574)	< 0.001
Birth length (cm)	48.00(45.00, 49.00)	48.00(45.00, 48.75)	46.00(42.00, 48.00)	0.001	47.00(45.00, 49.00)	48.00(44.00, 48.00)	46.00(40.00, 46.00)	< 0.001
Head circumference (cm)	33(32, 33)	33(32, 33)	33(31, 33)	0.015 0.169	33(32, 33)	33(32, 33)	32(32, 34)	0.046 0.007
Birth asphyxia								
No	555(74.8)	421(73.6)	134(78.8)		239(75.4)	184(72.2)	55(88.7)	
Yes	187(25.2)	151(26.4)	36(21.2)		78(24.6)	71(27.8)	7(11.3)	
Early-onset sepsis				< 0.001				<0.001
No	449(60.6)	387(67.7)	62(36.5)		203(64.0)	176(69.0)	27(43.5)	
Yes	293(39.5)	185(32.3)	108(63.5)		114(36.0)	79(31.0)	35(56.5)	
Apgar score at 1 min	10(10, 10)	10(10, 10)	10(9, 10)	< 0.001	10(10, 10)	10(10, 10)	10(9, 10)	< 0.001
Apgar score at 5 min	10(10, 10)	10(10, 10)	10(9, 10)	< 0.001	10(10, 10)	10(10, 10)	10(9, 10)	< 0.001
Apgar score at 10 min	10(10, 10)	10(10, 10)	10(9, 10)	< 0.001	10(10, 10)	10(10, 10)	10(10, 10)	< 0.001
Delivery and resuscitation								
Mode of delivery				0.125				0.458
No	172(23.2)	140(24.5)	32(18.8)		78(24.6)	65(25.5)	13(21.0)	
Yes	570(76.8)	432(75.5)	138(81.2)		239(75.4)	190(74.5)	49(79.0)	
Umbilical cord abnormalities				0.017				0.980
No	619(83.4)	467(81.6)	152(89.4)		256(80.8)	206(80.8)	50(80.6)	
Yes	123(16.6)	105(18.4)	18(10.6)		61(19.2)	49(19.2)	12(19.4)	
Postnatal age at NICU admission (days)	0.30(0.22, 0.38)	0.31(0.15, 0.37)	0.25(0.17, 0.42)	< 0.001	0.30(0.21, 0.37)	0.30(0.15, 0.30)	0.25(0.19, 0.43)	0.016
Perinatal interventions								
Antenatal corticosteroid exposure	3(3, 3)	3(3, 3)	3(3, 3)	0.337	3(3, 3)	3(3, 3)	3(3, 3)	0.842
Pulmonary surfactant use within 72 h				< 0.001				<0.001
No	676(91.1)	566(99.0)	110(64.7)		289(91.2)	254(99.6)	35(56.5)	
Yes	66(8.9)	6(1.0)	60(35.3)		28(8.8)	1(0.4)	27(43.5)	
Initial feeding method				0.499				0.029
No	594(80.1)	461(80.6)	133(78.2)		243(76.7)	202(79.2)	41(66.1)	
Yes	148(19.9)	111(19.4)	37(21.8)		74(23.3)	53(20.8)	21(33.9)	
Laboratory findings at admission								
White blood cell count (× 10^9^/L)	8.93(7.40, 10.86)	8.88(7.31, 10.75)	9.02(7.43, 11.46)	0.235	8.79(7.44, 10.66)	8.79(7.44, 10.66)	8.35(7.25, 10.00)	0.411
Neutrophil percentage (%)	76.10(71.00, 80.00)	76.00(71.00, 80.10)	76.70(71.00, 80.13)	0.079	76.00(71.50, 79.90)	76.10(71.70, 80.40)	75.55(71.48, 79.55)	0.590
Lymphocyte percentage (%)	17.90(14.58, 22.60)	18.10(14.60, 22.48)	17.50(14.00, 21.45)	0.075	18.40(14.55, 22.75)	18.00(13.10, 22.00)	18.95(16.35, 23.00)	0.174
Hemoglobin (g/L)	118(109, 126)	118(109, 126)	116(109, 126)	0.137	115 ± 14	115 ± 14	115 ± 14	0.850
Platelet count (× 10^9^/L)	202.00(165.00, 246.25)	202.50(165.00, 244.00)	198.50(165.00, 265.00)	0.893	208.00(168.00, 244.50)	208.00(168.00, 244.50)	206.00(157.00, 247.00)	0.582

1. Continuous variables are presented as median (IQR) for skewed data and mean ± SD for approximately normal data; categorical variables as n (%). Group comparisons used the Mann–Whitney U, independent samples *t*-test, or χ^2^/Fisher’s exact test, as appropriate. 2. Apgar shows a ceiling effect (many 9–10); hence we report median (IQR). Significant Mann–Whitney P-values may occur even when medians appear similar because the test compares rank distributions. For interpretability, the proportion with 1-min Apgar ≤ 6 is also reported. 3. In the table, “No/Yes” = absence/presence. Key mappings: Mode of delivery—No = Vaginal, Yes = Cesarean; Mode of conception—No = Natural, Yes = ART; Initial feeding (0–72 h)—No = Human milk only, Yes = Any formula; PS ≤ 72 h—No = Not given, Yes = Given (before first IMV); EOS ≤ 72 h—No = Absent, Yes = Present; IMV ≤ 72 h—No = Not meeting outcome, Yes = Endotracheal invasive ventilation ≥ 12 h within 72 h. Premature rupture of membranes was coded as a binary obstetric variable (Yes/No), rather than by duration in hours or days. Antenatal corticosteroid exposure was not analyzed by hours or days; instead, it was coded as an ordinal variable according to documented treatment status before delivery (1 = complete course, 2 = incomplete course, and 3 = no exposure). A complete course was defined as either two 12-mg intramuscular doses given 24 h apart or four 6-mg intramuscular doses given 12 h apart. Abnormal placental pathology referred to documented abnormal findings on placental pathology examination when placental pathology data were available; placental examination was not routinely performed in all preterm infants. Abnormal umbilical cord included documented nuchal cord, torsion, true knot, prolapse, edema, and related cord abnormalities. 4. Abbreviations: IMV, invasive mechanical ventilation; EOS, early-onset sepsis; PS, pulmonary surfactant.

In the training cohort, infants who required mechanical ventilation had a significantly lower gestational age than those who did not [median 34 weeks (IQR: 33–36) vs. 35 weeks (IQR: 34–36); *P* < 0.001]. Birth weights were also significantly lower in the mechanical ventilation group [2140 g (IQR: 1337–2140) vs. 2395 g (IQR: 1805–2395); *P* < 0.001]. Additionally, birth length, head circumference, and Apgar scores at all measured time points were significantly lower in the ventilation group (all *P* < 0.05). The prevalence of early-onset sepsis (EOS) (63.5% vs. 32.3%; *P* < 0.001) and pulmonary surfactant (PS) use (35.3% vs. 1.0%; *P* < 0.001) was significantly higher in the mechanical ventilation group than in the non-ventilation group.

Similar trends were observed in the validation cohort. In the validation cohort, infants who required mechanical ventilation also had significantly lower gestational age [34 weeks (IQR: 34–36) vs. 35 weeks (IQR: 34–36); *P* < 0.001] and birth weight [2115 g (IQR: 1795–2574) vs. 2410 g (IQR: 2000–2580); *P* < 0.001]. A significantly greater proportion of infants in the ventilation group had EOS (56.5% vs. 31.0%; *P* < 0.001) and received PS treatment (43.5% vs. 0.4%; *P* < 0.001). Maternal demographics, obstetric complications, mode of delivery, and admission laboratory results did not differ significantly between the two groups (all *P* > 0.05). These findings suggest that the need for mechanical ventilation is closely associated with perinatal characteristics and early clinical interventions in preterm infants.

### Screening of candidate variables

**FIGURE 1:**
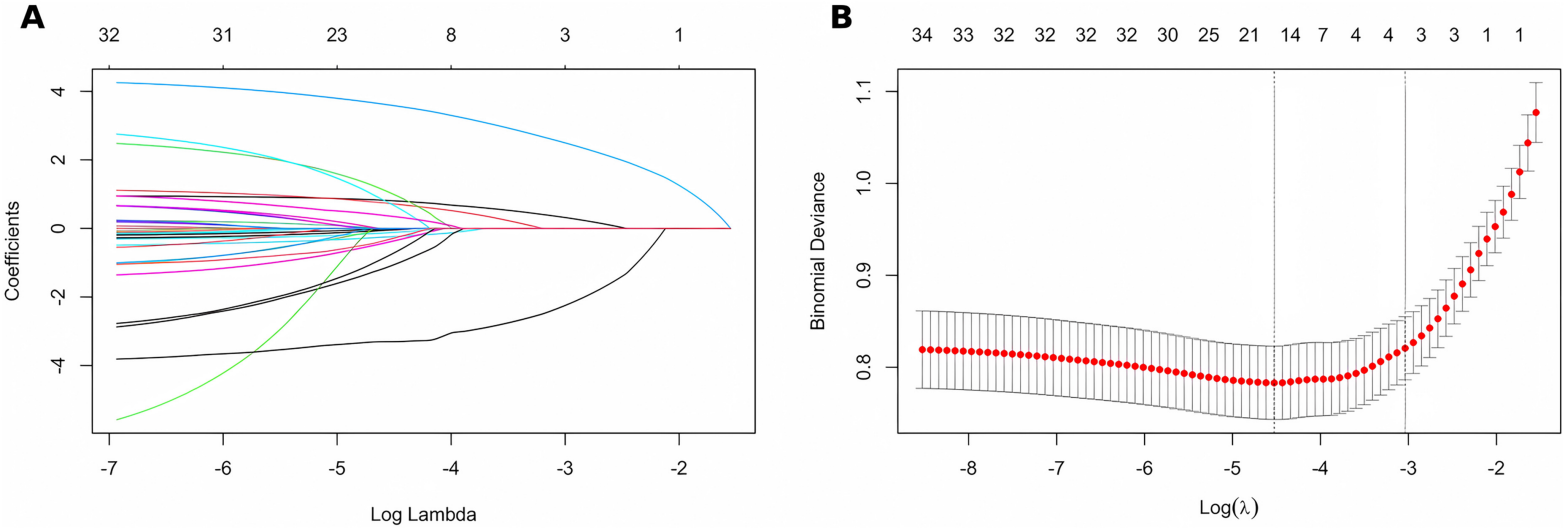
LASSO-based feature selection for the prediction of invasive mechanical ventilation within 72 h. **(A)** Coefficient profiles of the 36 candidate predictors entered into the LASSO model. **(B)** Twenty-fold cross-validation plot showing λmin (minimum cross-validation error) and λ1se (the largest λ within one standard error of the minimum). The λ1se criterion was selected, retaining three predictors with non-zero coefficients for final model construction.

Among 36 candidate predictors, several showed crude associations with the outcome in univariable analyses (Supplementary Table 2). Variables with *P* < 0.10 and those deemed clinically pertinent were entered into least absolute shrinkage and selection operator (LASSO) regression. Gestational age and birth weight were included in the candidate set because both were significantly associated with the outcome in univariable analyses. However, 20-fold cross-validation identified the λ_1s*e*_ penalty, which retained only three predictors for final model building: 1-min Apgar score, pulmonary surfactant (PS) within 72 h, and early-onset sepsis (EOS) (Figure 1).

### Multivariable logistic regression model

In the multivariable model (Table 2), each 1-point decrease in the 1-min Apgar score was associated with higher odds of invasive mechanical ventilation (IMV) within 72 h (OR 1.87, 95% CI 1.46–2.39, *P* < 0.001). No pulmonary surfactant within 72 h (vs. PS) was associated with higher odds of IMV (OR 50.0, 95% CI 20.41–125.00, *P* < 0.001), and No early-onset sepsis (vs. EOS) was likewise associated with higher odds (OR 2.46, 95% CI 1.57–3.88, *P* < 0.001). These ORs are re-expressed for interpretability (see Table 2 footnote); statistical significance is unchanged.

**TABLE 2:** Multivariable logistic regression analysis for predicting mechanical ventilation within 72 h after birth in the training cohort.

Variable	β	Odds ratio (95% CI)	*P*
Apgar score at 1 min	−0.626	0.535(0.418–0.684)	<0.001
Pulmonary surfactant use within 72 h	−3.909	0.020(0.008–0.049)	<0.001
Early-onset sepsis	−0.902	0.406(0.258–0.639)	< 0.001

1. Variables were selected based on least absolute shrinkage and selection operator (LASSO) regression and clinical relevance. 2. Results are presented as β coefficients, odds ratios (ORs) with 95% confidence intervals (CIs), and *P*-values. 3. All included predictors were statistically significant (*P* < 0.001) and used to construct the final prediction model. 4. MV, Mechanical ventilation; EOS, Early-onset sepsis; PS, Pulmonary surfactant.

### Nomogram construction

**FIGURE 2:**
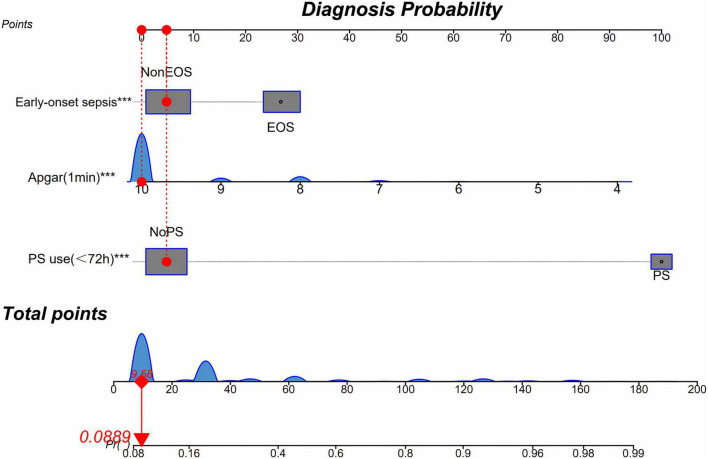
Nomogram for predicting invasive mechanical ventilation within 72 h in preterm infants. The nomogram incorporates 1-min Apgar score, early-onset sepsis (EOS), and pulmonary surfactant (PS) use within 72 h after birth. Points assigned to each predictor are summed to generate a total score, which corresponds to the predicted probability of invasive mechanical ventilation within 72 h. The outcome was defined as endotracheal invasive mechanical ventilation lasting ≥ 12 consecutive hours within the first 72 h after birth; CPAP/NIPPV and brief intubations solely for INSURE/LISA were not counted.

A nomogram was developed based on the multivariable logistic regression results to estimate the likelihood of mechanical ventilation within 72 h in preterm infants (Figure 2). The nomogram incorporated three predictors: 1-min Apgar score, pulmonary surfactant (PS) use within 72 h, and early-onset sepsis (EOS). This tool allows clinicians to rapidly estimate the individualized probability of mechanical ventilation based on the three predictor scores.

### Model discrimination ability assessment

**FIGURE 3:**
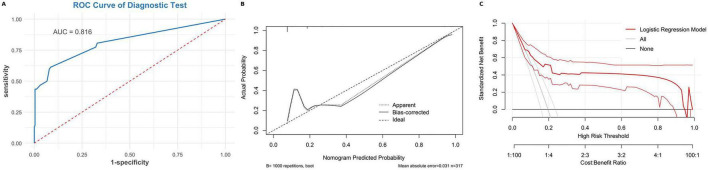
Discrimination, calibration, and clinical utility of the prediction model in the validation cohort. **(A)** Receiver operating characteristic (ROC) curve of the model in the validation cohort, with an area under the curve (AUC) of 0.816. **(B)** Calibration plot based on 1,000 bootstrap resamples. The dashed line indicates perfect calibration, the dotted line indicates apparent performance, and the solid line indicates bias-corrected performance. **(C)** Decision curve analysis (DCA) showing the net clinical benefit of the model across a range of threshold probabilities compared with the treat-all and treat-none strategies.

The model’s discriminative performance was assessed using receiver operating characteristic (ROC) curve analysis. In the validation cohort, the area under the ROC curve (AUC) was 0.816 (Figure 3A). At the optimal cutoff of 0.224, the sensitivity, specificity, and overall accuracy were 0.613, 0.914, and 0.855, respectively. The positive predictive value (PPV) and negative predictive value (NPV) were 0.633 and 0.907, respectively, indicating favorable predictive accuracy.

In the training cohort, the AUC reached 0.822 (Supplementary Figure 1A). At this threshold, the sensitivity, specificity, and accuracy were 0.594, 0.906, and 0.834, respectively. The PPV and NPV were 0.652 and 0.882, respectively. Consistent performance across both cohorts suggests that the model has adequate stability and generalizability.

### Model calibration capability assessment

Calibration curve analysis demonstrated that the model had strong predictive agreement with observed outcomes. In the validation cohort, the model showed excellent calibration (Figure 3B), with a Brier score of 0.096, Hosmer–Lemeshow χ^2^ = 2.52 (*P* = 0.28), calibration intercept = 0, slope = 1, and an expected-to-observed (E/O) ratio of 1—indicating no systematic over- or underestimation of risk. Calibration performance in the training cohort was similarly favorable (Supplementary Figure 1B), with a Brier score of 0.118, HL test *P* = 0.053, and an E/O ratio of 1. Calibration results across both cohorts support the accuracy and robustness of the model’s risk estimation.

### Model clinical utility assessment

Clinical utility was evaluated using decision curve analysis (DCA). In the validation cohort, the model yielded a clearly superior net benefit compared with “treat-all” and “treat-none” strategies when the predicted probability threshold ranged from 0.1 to 0.7 (Figure 3C), indicating strong clinical applicability. A similar pattern was observed in the training cohort (Supplementary Figure 1C), further supporting the model’s clinical utility.

### Sensitivity analysis—culture-proven EOS (validation cohort)

To address potential misclassification of clinically diagnosed EOS, we performed a sensitivity analysis in which EOS was restricted to culture-positive cases only. Six infants with missing/indeterminate cultures were excluded from the validation set [*n* = 311; culture-positive 15/311 (4.8%)]. Using predicted probabilities from the refit three-predictor model (same train–validation split), the validation AUC was 0.830 (95% CI 0.768–0.891) versus 0.816 in the main analysis (ΔAUC = 0.014), with Brier score = 0.081. ROC curves were largely overlapping, and decision-curve analysis showed higher standardized net benefit than “treat-all” or “treat-none” across thresholds ≈0.05–0.70 (Supplementary Table 3 and Supplementary Figure 2). These results indicate stable discrimination and clinical utility under a culture-proven EOS definition.

### Interaction effect analysis

**FIGURE 4:**
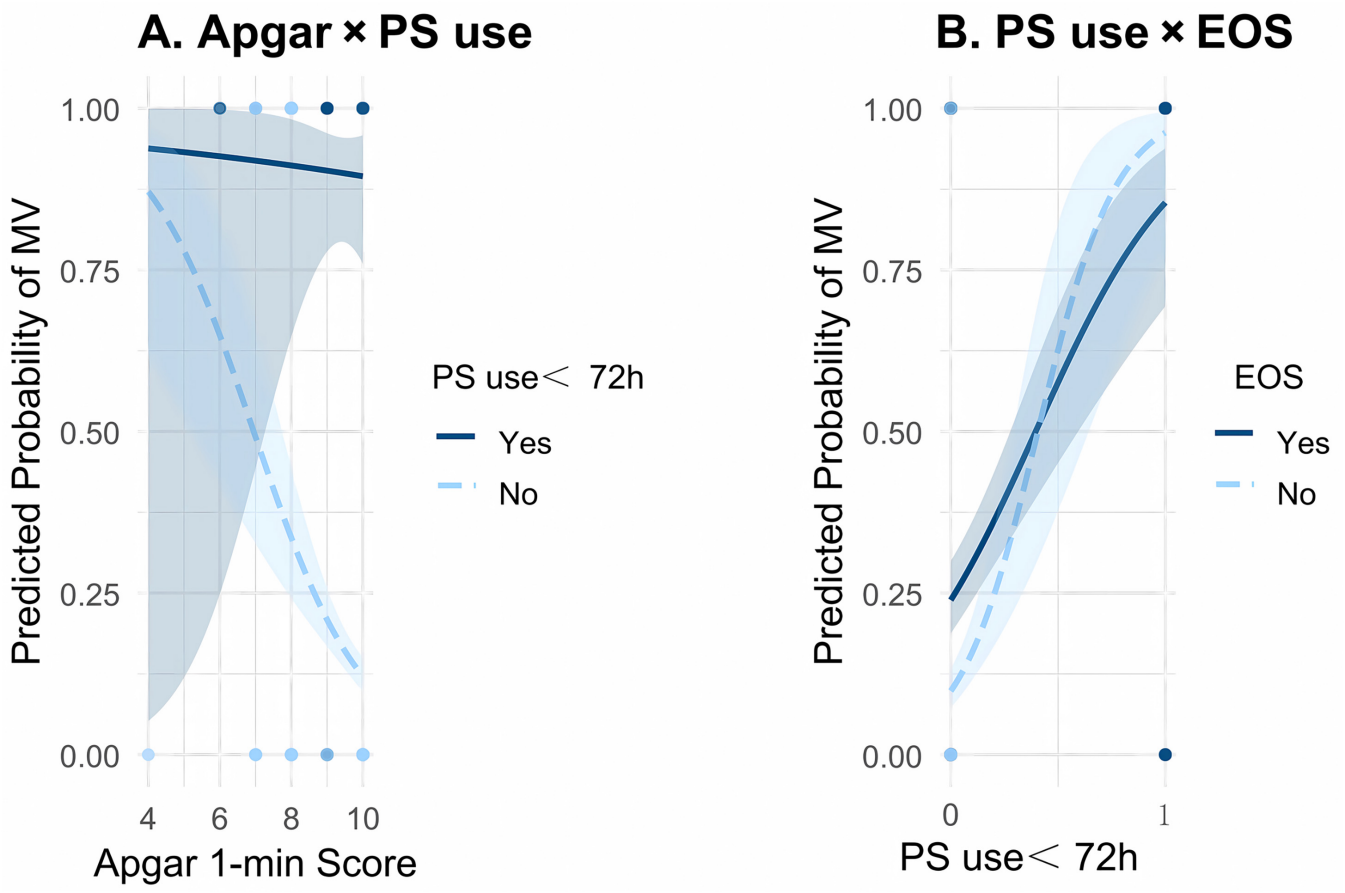
Interaction effects among key predictors for invasive mechanical ventilation risk. **(A)** Interaction between 1-min Apgar score and pulmonary surfactant (PS) use within 72 h. **(B)** Interaction between PS use and early-onset sepsis (EOS). The curves represent model-based predicted probabilities of invasive mechanical ventilation within 72 h, and the shaded areas indicate 95% confidence intervals.

We explored potential interaction effects among the model’s key predictors to assess whether their combined influence modified the risk of mechanical ventilation. Interaction analysis revealed no significant effect between 1-min Apgar score and PS use (β = 0.553; *P* = 0.295; Figure 4A). However, a trend suggested that neonates with low Apgar scores who did not receive PS had an elevated risk of requiring mechanical ventilation. A significant interaction was observed between PS use and EOS (β = −2.531; *P* = 0.028; Figure 4B), indicating that EOS substantially modified the association between PS and the need for mechanical ventilation, suggesting that PS may have a protective effect against ventilation in the presence of EOS. These findings suggest potential physiological regulatory mechanisms linking the predictor variables. These interactions enhance the model’s clinical interpretability and practical relevance.

## Discussion

### Main findings

In this single-center retrospective cohort of 1,059 preterm infants, we found that the receipt of invasive mechanical ventilation (MV) within 72 h after birth could be accurately predicted using three perinatal indicators: 1-min Apgar score, pulmonary surfactant (PS) use within 72 h, and early-onset neonatal sepsis (EOS). The resulting nomogram showed robust performance in the validation cohort (AUC = 0.816; Brier = 0.096; E/O = 1), with sustained net clinical benefit observed across the 0.1–0.7 probability threshold range. Compared with traditional multivariable scoring systems (e.g., SNAP-II) and recent “black-box” machine learning models, the nomogram achieved comparable or even superior discrimination using only three variables. It also offers high interpretability and ease of integration into electronic medical record (EMR)-based clinical decision support systems (CDSS) (5, 13, 14). Overall, the model presents a practical, efficient, and low-cost approach to risk stratification for guiding early respiratory support decisions in the NICU.

### Interpretation of results and potential mechanisms

A low 1-min Apgar score reflects perinatal hypoxia and cardiopulmonary depression, potentially leading to rapid surfactant depletion and reduced respiratory drive. A multicenter prospective study of 7,566 infants born at < 28 weeks’ gestation showed that lower 1-min Apgar scores were associated with increased risks of MRI-detected white-matter injury and the need for invasive respiratory support (15). Similarly, among 413 infants born at < 32 weeks, the BPD group had significantly lower 1-min Apgar scores than controls (6 vs. 8; *P* < 0.001) (16). In our model, each 1-point decrease in the 1-min Apgar score was associated with higher odds of IMV within 72 h (OR≈1.87), underscoring the clinical utility of the 1-min Apgar score as an early predictive signal of adverse respiratory outcomes.

Pulmonary surfactant (PS) administration within 72 h is often used at the bedside as a proxy for RDS severity. The 2022 European Consensus Guidelines on RDS recommend PS when FiO_2_ > 0.30 or upon CPAP failure, both reflecting greater alveolar instability and respiratory effort (1). In our model, lack of PS within 72 h (vs PS) was associated with higher odds of IMV within 72 h (OR ≈ 50.0, 95% CI 20.41–125.00), which is consistent with practice patterns whereby sicker infants are prioritized for early surfactant. This association aligns with evidence that, even with LISA strategies, about 20% of preterm infants require intubation within 7 days of initial PS, and under conventional approaches ∼30–40% progress to IMV (17). We interpret the PS coefficient as predictive rather than causal, recognizing pathway dependence and potential indication bias.

The link between EOS and mechanical ventilation has been attributed to inflammatory cascades: endotoxin–LBP complexes rapidly trigger pro-inflammatory responses (e.g., IL-6, TNF-α), which can impair alveolar epithelial integrity and suppress type II pneumocyte secretion, thereby reducing lung compliance and oxygenation reserve (18). A recent review emphasized that EOS-related immune “overactivation–exhaustion” may compromise spontaneous respiratory function, making early mechanical ventilation a frequent rescue intervention in some settings (19). In our multivariable model, the absence of EOS (vs. EOS) was associated with higher odds of IMV within 72 h (OR≈2.46); equivalently, EOS was associated with lower odds of IMV. Given diagnostic heterogeneity and practice patterns in retrospective cohorts, we interpret this coefficient as predictive rather than causal, and acknowledge that indication/diagnostic bias may influence the observed direction. Our prior EOS nomogram and the present IMV-risk model address different clinical questions and outcomes, although they were derived from the same underlying clinical cohort framework. Thus, overlap in predictors should be interpreted as reflecting shared early neonatal risk signals rather than causal equivalence between the two models. The association between EOS and respiratory support needs has also been described in previous studies; however, in retrospective cohorts, the observed magnitude and direction may be influenced by diagnostic heterogeneity and local treatment practices.

Interaction analysis further highlighted the physiological interdependence among the three perinatal factors:

Apgar × PS: Among newborns without PS treatment, each 1-point decrease in Apgar score was linked to a markedly higher risk of mechanical ventilation within 72 h (β = 0.553). This suggests that the co-occurrence of hypoxic stress and surfactant deficiency may synergistically aggravate the decline in lung compliance.

PS × EOS: In EOS-positive neonates, early PS administration significantly reduced the risk of mechanical ventilation (β = −2.531, *P* = 0.028), likely mitigating inflammation-driven alveolar dysfunction. These findings align with evidence from animal studies. In a ventilated neonatal rabbit model, Xu et al. showed that exogenous PS not only enhanced lung compliance but also markedly suppressed pulmonary GBS proliferation and translocation, ultimately reducing the intensity of respiratory support required (20).

In summary, the three perinatal factors independently influence early ventilation needs, while their interactions reveal a converging mechanistic pathway—hypoxia, inflammation, and surfactant imbalance—that may underlie the heterogeneity of neonatal respiratory failure and support tailored clinical decision-making.

### Clinical application value

The nomogram enables early postnatal individualized risk assessment during the first 72 h after birth and may be integrated into NICU respiratory management workflows once the required clinical information becomes available. Because EOS cannot be definitively classified in the first minutes of life, the model should be interpreted as an early postnatal rather than a strict delivery-room prediction tool. For infants with a predicted risk ≥ 0.70, tracheal intubation and infusion pump setup should be completed within 30 min. Those with a predicted probability between 0.30 and 0.69 may initially receive guideline-concordant non-invasive respiratory support with close re-evaluation at 6 h. Infants with a predicted risk < 0.30 should continue with routine monitoring. Based on the validation cohort, this stratification approach may help avoid unnecessary intubation in approximately 60% of low-risk infants while maintaining a missed diagnosis rate below 12%. A multicenter prospective cohort study found that each additional day of initial invasive mechanical ventilation nearly doubled the risk of moderate-to-severe BPD (aOR ≈ 1.97), highlighting the importance of minimizing invasive ventilation early (21). Economic modeling suggests that non-invasive strategies, such as high-flow nasal cannula, may reduce hospitalization costs by approximately $2,000 per infant. A 10% reduction in annual ventilation rates could yield cost savings of $200,000 to $250,000 per hospital per year (22). Respiratory-support decisions informed by the model should remain consistent with contemporary RDS guidelines, and HFNC should not be interpreted here as routine primary support. The three predictive variables are documented during the early perinatal and postnatal course and can be incorporated into electronic medical record-based clinical decision support once the relevant information becomes available. These variables can be seamlessly integrated into clinical decision support systems (CDSS) via HL7 or RESTful interfaces. A recent implementation study demonstrated that embedding real-time decision prompts reduced extubation failure rates from 10.3 to 2.3% and markedly enhanced adherence to clinical guidelines (23). Thus, the model offers unique advantages—immediacy, low cost, and strong interpretability—making it a practical tool for precision respiratory management in NICUs.

### Comparison with existing ratings/models

SNAP-II and SNAPPE-II are the most commonly used tools to evaluate respiratory outcomes in extremely preterm infants in the NICU. Both scores require 6–8 physiological and laboratory variables for calculation. These data typically become available only 12–24 h after birth, limiting their utility for early decision-making. A retrospective study of 192 infants with NRDS conducted by Yan et al. reported an AUC of only 0.730 for SNAP-II in predicting in-hospital mortality, reflecting limited discriminative performance (24). In 2023, Kim et al. developed a bidirectional long short-term memory (LSTM) deep learning model based on 62 monitoring and EMR features collected within 24 h, achieving an internal validation AUC of 0.861, higher than traditional random forest and XGBoost models. However, the algorithm remains a black box, lacks external validation, and raises concerns regarding transparency, clinical interpretability, and real-world applicability (5). In 2024, Lei et al. used data from 705 NRDS patients in the MIMIC-IV database to build a nomogram integrating 11 perinatal and blood gas indicators, achieving a validation AUC of 0.924. However, the model’s heavy reliance on laboratory data, implementation complexity, and single-center origin necessitate further evaluation of its generalizability across diverse clinical settings (25). Compared to these models, our study achieved an AUC of 0.816 in the validation cohort using only three perinatal indicators readily available on the day of birth: 1-min Apgar score, early-onset sepsis, and pulmonary surfactant use within 72 h. The nomogram visually presents individual risk levels while balancing simplicity, interpretability, and potential generalizability. These findings support the development of a streamlined decision support tool for early respiratory management in NICUs.

### Research advantages

This study offers notable strengths in both methodological rigor and clinical applicability. First, the study features a robust sample size (*n* = 1,059) and an event-to-variable ratio of ∼70:1, well above the TRIPOD threshold of 10 events per predictor. Model performance was assessed using multiple metrics—training-validation stratification, Brier score, Hosmer–Lemeshow test, and decision curve analysis—to ensure robustness. These evaluations confirmed the model’s stability and predictive reliability (26). Second, the model relies exclusively on three real-time perinatal indicators, ensuring simplicity and minimizing data loss. This design facilitates seamless integration into electronic health record (EHR) systems. Third, the model provides calibration and decision curves, along with interaction analyses that enhance its explanatory power. These features improve both the biological interpretability and clinical credibility of the model. The study adheres strictly to TRIPOD reporting standards and incorporates the PROBAST quality assessment framework, ensuring transparent, rigorous, and reproducible methodology. This foundation supports future multicenter external validation studies.

### Limitations

This study has several limitations. First, it was a single-center retrospective cohort; local practice patterns (e.g., thresholds for EOS diagnosis/antibiotics, use of LISA/MIST, and ventilation protocols) may limit transportability. External validation across time and settings is warranted, consistent with TRIPOD guidance (27). Second, we deliberately excluded infants with chorioamnionitis to reduce exposure misclassification and spectrum bias; the nomogram is therefore intended for CA-negative settings, and validation in cohorts with higher CA prevalence is needed (8, 9). Third, the observed EOS rate was higher than that reported in multinational surveillance studies, likely reflecting our unit’s low threshold for empirical antibiotics during delivery-room resuscitation; similar clinical-EOS inflation has been reported in extremely preterm cohorts (28, 29). Importantly, a sensitivity analysis restricting EOS to culture-proven cases yielded similar validation performance (AUC 0.830 vs. 0.816; ΔAUC = 0.014) and overlapping calibration/decision-curve profiles (Supplementary Table 3 and Supplementary Figure 2), supporting robustness to EOS definition. Fourth, pulmonary-surfactant administration is pathway-dependent; we interpret its coefficient as predictive rather than causal, noting that brief intubations solely for surfactant (INSURE/LISA) were not counted as events. Fifth, the model used static perinatal variables and did not incorporate continuous physiologic time-series (e.g., SpO_2_ variability, apnea burden); future work should evaluate whether such signals improve early risk stratification and dynamic alerting. Sixth, the endpoint was binary IMV (endotracheal ventilation ≥ 12 h within 72 h); we did not model duration or mode beyond this threshold, nor long-term outcomes (e.g., VILI, BPD). Seventh, the decision threshold (0.224) was selected by the Youden index; clinical adoption will require site-specific recalibration and cost/benefit-aware thresholding. In addition, because EOS cannot be definitively classified in the first minutes of life, the model should be interpreted as an early postnatal risk-stratification tool rather than a strict delivery-room prediction model. Finally, by excluding infants who died ≤ 72 h, the model does not apply to ultra-high-risk early deaths; limited availability of inflammatory biomarkers (e.g., IL-6, PCT) may have left residual confounding.

### Future research directions

Future efforts will advance along three parallel and interconnected paths: external validation, model refinement, and clinical impact evaluation. We plan to collaborate with at least five tertiary NICUs to conduct dual geo-temporal external validation in accordance with TRIPOD-Cluster guidelines. This multicenter validation will systematically capture variation in infection patterns and ventilation practices to assess the model’s cross-site discrimination, calibration, and need for recalibration (27). Building on this, we aim to embed a “sliding window re-learning plus federated learning” mechanism into hospital EMRs to integrate continuous signals, such as SpO_2_ variability, apnea index, and inflammatory/metabolic biomarkers, in real time. This will enable the development of a multimodal dynamic model to evaluate its added value for early ventilation decisions and its generalizability across centers. Finally, we will adopt a stepped-wedge or pre-post quasi-randomized implementation design to monitor mechanical ventilation rates, BPD incidence, and hospitalization costs following model integration. A recent Pediatrics study demonstrated that incorporating a real-time extubation success calculator into the EMR reduced reintubation rates in extremely preterm infants from 10.3 to 2.3%—a relative reduction of 78%—and shortened the average ventilation duration by 35%. This serves as a compelling example for evaluating cost-effective adoption strategies (23).

## Conclusion

This study developed a visual nomogram based on three early perinatal and early neonatal indicators—1-min Apgar score, pulmonary surfactant use within 72 h, and early-onset neonatal sepsis—that enables accurate, robust, and interpretable prediction of mechanical ventilation risk within the first 72 h of life in preterm infants (AUC = 0.816 in the validation cohort; good calibration and net benefit). The model may be integrated into NICU electronic medical record systems to support early postnatal respiratory risk stratification once the required clinical information has become available. In this context, EOS should be interpreted as a predictive marker within an IMV-risk model rather than as direct evidence of a causal pathway. Future multicenter external validation and digital re-learning will further enhance the model’s translational potential for precision respiratory management and NICU resource optimization.

## Data Availability

The datasets presented in this study can be found in online repositories. The names of the repository/repositories and accession number(s) can be found at: All de-identified datasets and full analysis code are openly available at Zenodo, doi: 10.5281/zenodo.17272050.
