# Supplementary material for: A three-factor nomogram predicts the use of invasive mechanical ventilation within 72 h in preterm infants
Source: Front Med (Lausanne). 2026 Apr 29;13:1722043. doi: 10.3389/fmed.2026.1722043 (PMC13168056; doi:10.3389/fmed.2026.1722043)
Supplement: Supplementary file 1 [file Data_Sheet_1.docx]

Supplementary Material

Supplementary Table 1. Performance Comparison of Five Imputed Datasets: AUC, Accuracy, Sensitivity, and Specificity

| Data Set | AUC | Accuracy | Sensitivity | Specificity |
| --- | --- | --- | --- | --- |
| Data1 | 0.8193 | 0.839 | 0.592 | 0.909 |
| Data2 | 0.8199 | 0.840 | 0.597 | 0.909 |
| Data3 | 0.8244 | 0.841 | 0.601 | 0.909 |
| Data4 | 0.8238 | 0.839 | 0.592 | 0.909 |
| Data5* | 0.8243 | 0.841 | 0.601 | 0.909 |

Supplementary Table 1 presents the model performance (AUC, accuracy, sensitivity, specificity) across five imputed datasets generated from the original data. Given the negligible differences in performance across datasets, **Dataset 5** (denoted with an asterisk) was randomly selected for further analysis.

Supplementary Table 2. Univariable logistic regression analysis of candidate predictors for mechanical ventilation within 72 hours after birth in the training cohort.

| **Variable** | **β** | **Odds ratio(95%CI)** | ***P*** |
| --- | --- | --- | --- |
| **Maternal demographics** |  |  |  |
| Maternal age (years) | -0.013 | 0.987(0.947–1.028) | 0.525 |
| Maternal occupation | 0.241 | 1.272(0.896–1.806) | 0.178 |
| Maternal smoking | 0.402 | 1.495(0.324–6.888) | 0.606 |
| Gravidity (n) | 0.011 | 1.011(0.813–1.256) | 0.922 |
| Parity (n) | 0.062 | 1.064(0.872–1.298) | 0.542 |
| Mode of conception | -0.132 | 0.876(0.533–1.441) | 0.602 |
| **Maternal complications** |  |  |  |
| Hypertensive disorders in pregnancy | -0.170 | 0.843(0.580–1.227) | 0.373 |
| Gestational diabetes mellitus | -0.126 | 0.881(0.606–1.281) | 0.508 |
| Intrapartum fever | -0.117 | 0.889(0.283–2.794) | 0.841 |
| Premature rupture of membranes | 0.318 | 1.374(0.924–2.045) | 0.117 |
| Placenta previa | 0.640 | 1.897(0.651–5.528) | 0.241 |
| Placental abruption | -0.338 | 0.713(0.291–1.750) | 0.460 |
| Abnormal placental pathology | 0.325 | 1.384(0.856–2.237) | 0.185 |
| **Perinatal characteristics** |  |  |  |
| Singleton pregnancy | -0.279 | 0.756(0.525–1.088) | 0.133 |
| Neonatal sex | 0.251 | 1.286(0.906–1.824) | 0.159 |
| Gestational age (weeks) | -0.298 | 0.742(0.681–0.809) | < 0.001 |
| Additional gestational days | -0.012 | 0.988(0.906–1.079) | 0.795 |
| Birth weight (g) | -0.001 | 0.999(0.999–1.000) | < 0.001 |
| Birth length (cm) | -0.084 | 0.920(0.880–0.961) | < 0.001 |
| Head circumference (cm) | -0.146 | 0.864(0.790–0.944) | 0.001 |
| Birth asphyxia | 0.289 | 1.335(0.884–2.016) | 0.169 |
| Early-onset sepsis | -1.293 | 0.274(0.192–0.393) | < 0.001 |
| Apgar score at 1 minute | -0.835 | 0.434(0.350–0.538) | < 0.001 |
| Apgar score at 5 minutes | -1.143 | 0.319(0.227–0.449) | < 0.001 |
| Apgar score at 10 minutes | -1.258 | 0.284(0.197–0.409) | < 0.001 |
| **Delivery & resuscitation** |  |  |  |
| Mode of delivery | -0.335 | 0.716(0.466–1.099) | 0.126 |
| Umbilical cord abnormalities | -0.641 | 0.527(0.309–0.897) | 0.018 |
| Postnatal age at NICU admission (days) | -3.782 | 0.023(0.005–0.105) | < 0.001 |
| **Perinatal interventions** |  |  |  |
| Antenatal corticosteroid exposure | -0.265 | 0.767(0.488–1.205) | 0.250 |
| Pulmonary surfactant use within 72 hours | -3.941 | 0.019(0.008–0.046) | < 0.001 |
| Initial feeding method | -0.144 | 0.866(0.569–1.316) | 0.499 |
| **Laboratory findings at admission** |  |  |  |
| White blood cell count (×10⁹/L) | 0.019 | 1.019(0.976–1.063) | 0.392 |
| Neutrophil percentage (%) | 0.020 | 1.020(0.997–1.043) | 0.094 |
| Lymphocyte percentage (%) | -0.027 | 0.973(0.947–1.000) | 0.050 |
| Hemoglobin (g/L) | -0.009 | 0.991(0.980–1.003) | 0.132 |
| Platelet count (×10⁹/L) | 0.001 | 1.001(0.998–1.004) | 0.385 |

**Notes:**1. Results are based on univariable logistic regression models with mechanical ventilation within 72 hours as the binary outcome variable.2. Values are presented as β coefficients, odds ratios (ORs) with 95% confidence intervals (CIs), and P values.3. OR < 1 indicates a protective association with mechanical ventilation, whereas OR > 1 indicates increased risk.4. Variables with *P* < 0.05 were considered statistically significant and selected for further screening.5. MV: Mechanical ventilation; EOS: Early-onset sepsis; PS: Pulmonary surfactant; NICU: Neonatal intensive care unit.

Supplementary Table 3. Culture-proven–only EOS sensitivity analysis (validation cohort)

| **Metric** |  | **Value** |
| --- | --- | --- |
| Cohort size (analyzed), n |  | 311 |
| Excluded for no/indeterminate culture, n |  | 6 |
| Events (culture-positive), n (%) |  | 15 (4.8%) |
| AUC (DeLong) |  | 0.830（0.768-0.891） |
| Brier score |  | 0.081 |

Notes: EOS definition: EOS counted only when culture-proven (positive blood/sterile-site culture ≤72 h). Cohort: Validation cohort n=311 after excluding 6 infants with missing/indeterminate cultures; culture-positive 15/311 (4.8%). Evaluation: Predicted probabilities were obtained from the training-set sensitivity model (same 3 predictors) and evaluated in the validation cohort. Metrics: AUC 95% CIs by DeLong; Brier score on a 0–1 scale. Outcome: Invasive mechanical ventilation via endotracheal tube, ≥12 h within 72 h; CPAP/NIPPV and brief INSURE/LISA not counted. Abbreviations: AUC, area under the receiver-operating characteristic curve; IMV, invasive mechanical ventilation.

Supplementary Figure 1. Discrimination, calibration, and decision curve analysis of the predictive model in the training cohort.


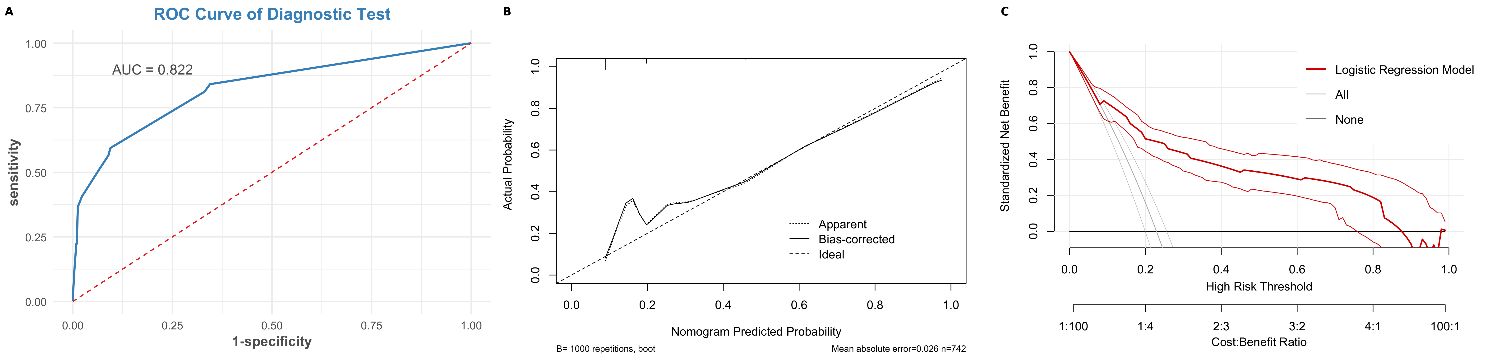

Note:(A) Receiver operating characteristic (ROC) curve of the predictive model in the training cohort. The area under the curve (AUC) was 0.822.
(B) Calibration curve with 1,000 bootstrap resamples. The dashed line represents perfect prediction; the dotted and solid lines represent the apparent and bias-corrected performance, respectively.
(C) Decision curve analysis (DCA) showing the net clinical benefit of using the model across a range of threshold probabilities, compared to “treat-all” and “treat-none” strategies. Outcome: invasive mechanical ventilation via endotracheal tube, ≥12 h within 72 h after birth; CPAP/NIPPV and brief INSURE/LISA intubations not counted.

Supplementary Figure 2. Decision-curve analysis for the culture-proven EOS sensitivity model (validation cohort)

**
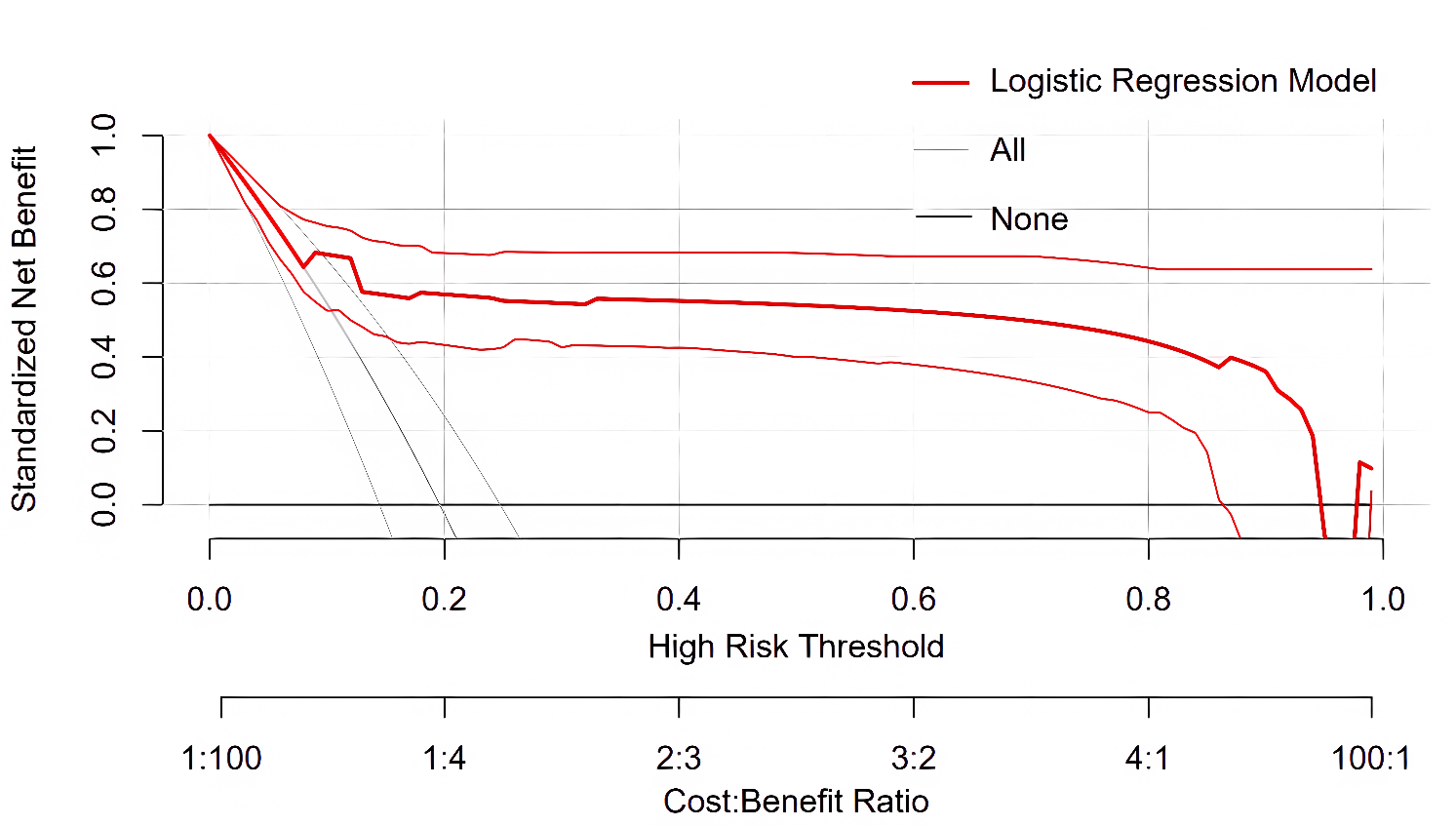
**

Note：Predicted probabilities were taken from the training-set sensitivity model and evaluated in the validation cohort (n=311; 15 culture-positive EOS; 6 excluded for missing/indeterminate cultures). Red = logistic model; grey = treat-all; black = treat-none. Standardized net benefit is shown across thresholds 0.05–0.70 (bootstrap 200 resamples for CIs). The model yields higher net benefit than either comparator across low-to-moderate thresholds. Outcome: Invasive mechanical ventilation (endotracheal, ≥12 h within 72 h; CPAP/NIPPV and brief INSURE/LISA not counted).
